# A new novel method for assessing intracranial pressure using non-invasive fundus images: a pilot study

**DOI:** 10.1038/s41598-020-70084-0

**Published:** 2020-08-03

**Authors:** Mikkel Schou Andersen, Christian Bonde Pedersen, Frantz Rom Poulsen

**Affiliations:** 1 Department of Neurosurgery, Odense University Hospital, 5000 Odense, Denmark; 2 Clinical Institute, University of Southern Denmark, Odense, Denmark; 3 BRIDGE (Brain Research - Inter Disciplinary Guided Excellence), University of Southern Denmark, Odense, Denmark

**Keywords:** Eye manifestations, Neurological manifestations

## Abstract

Arteriole and venule diameter ratio (A/V-ratio) can be measured using fundus photography. In this pilot study, we correlated changes in the intracranial pressure with the diameter of vessels of the retina. We investigated whether increased intracranial pressure (ICP) was reflected in a measurable and quantifiable distention of the venule diameter, leading to a decreased A/V-ratio. This was demonstrated by assessment of the A/V-ratio in patients already undergoing conventional ICP monitoring with a cerebral intraparenchymal pressure monitor. Our method shows a correlation between A/V ratio and ICP and suggests an easily obtainable and usable point-of-care (POC), non-invasive method to estimate the intracranial pressure without the necessity of mydriatic drugs. Furthermore, the sensitivity/specificity analysis with a cut-off of < 0.8015 A/V-ratio, showed a sensitivity of 94% [85–98%] and a specificity of 50% [34–66%] with a positive likelihood ratio of 9.0. This means that in a clinical setting there is a 94% chance of correctly identifying individuals with ICP ≥ 20 mmHg.

## Introduction

It is well-known that intracranial pressure (ICP) ranges from 5–15 mmHg at rest in a healthy adult^1,2^. The threshold value for intracranial hypertension varies not only throughout the literature but also in clinical practice. In general, ICP > 20 mmHg is considered pathological and often requires treatment^3–6^. ICP monitoring is considered paramount in diseases like intracranial hemorrhages, traumatic brain injury, subarachnoid hemorrhages, malignant infarction, cerebral edema and infections of the central nervous system to optimize treatment of secondary brain injuries and thereby improve outcome^7^.

Neuromonitoring still heavily relies on invasive methods such as parenchymal or ventricular neuromonitoring (external ventricular drain, EVD), both placed through a cranial burr hole^8^. Invasive procedures, however, carry the risk of bleeding^9,10^, infection^11^ and brain damage. Furthermore, invasive intracranial pressure tools are prone to device failure due to baseline drift of the probe in the tissue and breakage of equipment^10,12,13^, and the invasive nature of reliable ICP-monitoring equipment makes them unsuitable for usage in settings outside neurosurgical intensive care units.

To date, the most promising non-invasive ICP measuring methods studied are pupillometry ^14^ and optic nerve sheath diameter^15^. However, they have not been validated for daily clinical use and are prone to a high degree of inter-observer variations^16^. There is therefore a need for further research in other non-invasive intracranial pressure modalities.

In the United States and Europe, the incidence of traumatic brain injury (TBI) is 500/100,000 and more than 200/100,000 patients are admitted due to TBI related incidence in Europe^17,18^. A quick, non-invasive and easy-to-use screening tool for intracranial pressure would be of great benefit in such and other cases, where elevated intracranial pressure can be a potential hazard.

A relationship between acute elevation in ICP and retinal venule dilation was shown in animal models in the 70′s^19,20^. ICP affects intracranial arteries and venules and thereby influences the draining and feeding vessels to the eye, which in turn under higher ICP can cause papilledema by retrobulbar compression. Guidoboni et al.^21^ has published a theoretical work, which describes mathematically the association between vessel diameter, intraocular pressure and optic nerve tissue pressure and by that ICPs involvement. Furthermore, the intracranial hypertension treatment trial (2015) reported a decreasing retinal A/V-ratio in correlation with the severity of papilledema, which suggests an association between prolonged increased ICP and A/V-ratio^22^.

We here propose a novel, cheap, fast and easy measurement using non-invasive fundoscopy on non-dilated pupils to assess ICP by using retinal A/V-ratio. (ClinicalTrials.gov ID NCT03382860).

## Materials and methods

### Study design and population

The study was an observational, prospective and single-blinded proof-of-concept study of patients from a neurosurgical department investigated for suspected normal pressure hydrocephalus (NPH) or ventriculoperitoneal (VP) shunt dysfunction. Inclusion criteria were: 18 years or older and competent, informed consent and indication for intraparenchymal, intraventricular or lumbar intracranial pressure (ICP) monitoring. All experiment protocols were approved by The Regional Committees on Health Research Ethics for Southern Denmark, which is a part of the ethics committee system in Denmark (S-20170038). The Regional Committees on Health Research Ethics for Southern Denmark is based on the second Declaration of Helsinki and we confirm all methods were carried out in accordance with those guidelines and regulations.

### Demographics and clinical data description

All demographic and clinical data were either collected from the patient’s medical records or bedside. Data collected included: age, gender, blood pressure at time of data collection, past medical history, medication, smoking and indication for ICP monitoring.

### Fundus camera and experimental setup

**Figure 1:**
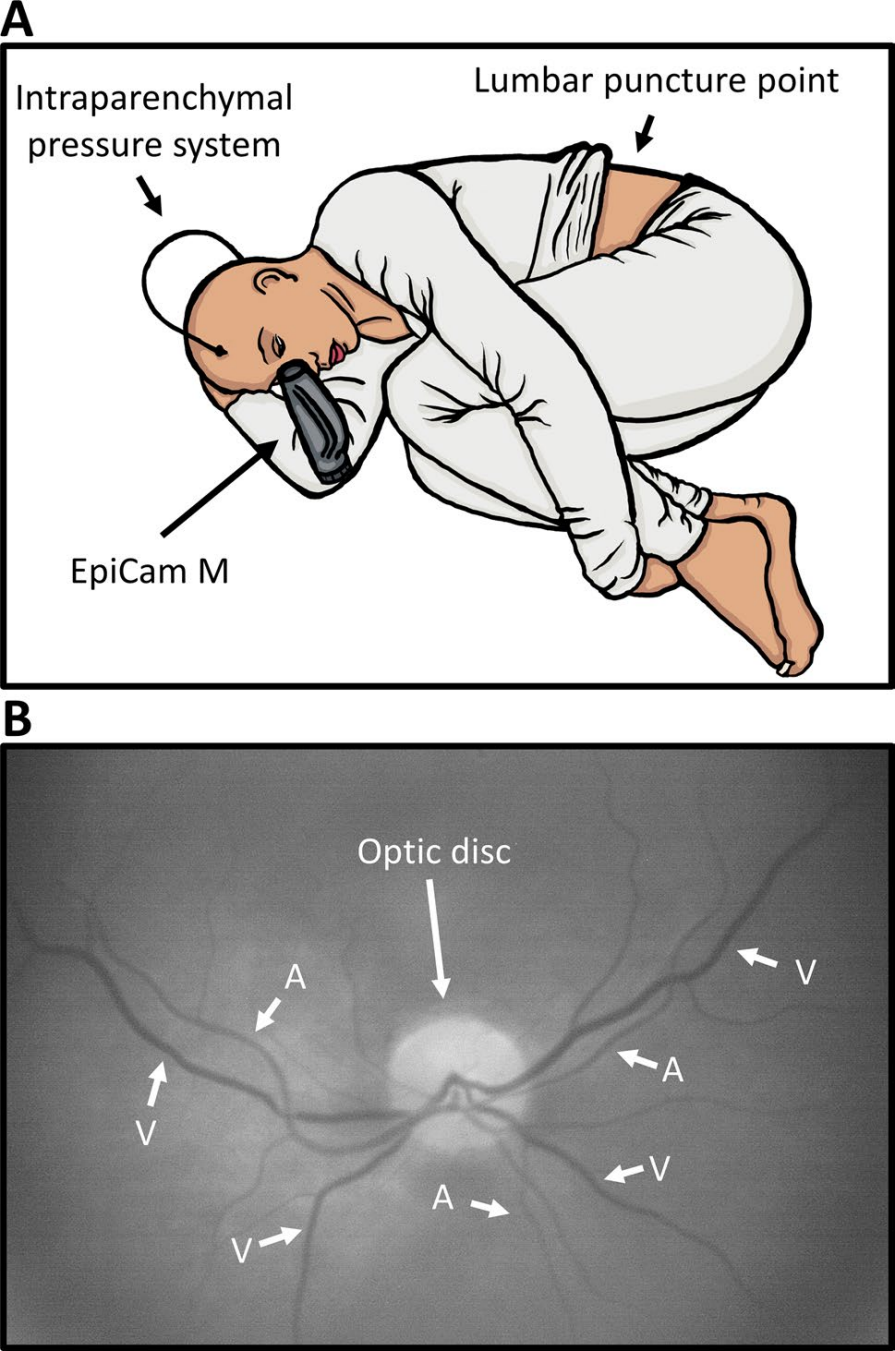
Experimental Setup and image. (**A**) The experimental setup. The EpiCam M is a hand-held digital retinal fundus camera, which is aimed directly at the pupil, from where the vessels can be visualized. This figure shows a schematic setup of the experimental setup. The video capturing occurs during infusion test through a needle in the lumbar region. (**B**) The optic disc and vessels. To distinguish arterioles and venules it is important to know two things: 1. Arterioles never cross arterioles and venules never cross venules and 2. Venules are generally larger and darker than arterioles. If the images are of high enough quality one can observe pulsation through the arteriole and thereby making proper vessel identification easy.

An EpiCam M (Epipole Ltd., Rosyth, UK), a handheld retinal fundus camera with a monochromatic image sensor, was used. The EpiCam is a CE marked Class I medical device and complies with ISO 10,940:2009, the international standard for retinal fundus cameras. The camera can correct focus in excess of ± 15 diopters. Mydriasis is not necessary. EpiCam viewer software version 2.1.1 (Epipole Ltd., Rosyth, UK) was used to capture the videos. Figure 1A shows experimental setup with the EpiCam M and Fig. 1B shows an example of an image that met the quality parameters. EpiCam viewer video analysis was performed with ICP and patient data blinded to the observer.

### Intracranial pressure monitoring

All videos were recorded with patients in a horizontal, left side position (Fig. 1A). Patients with suspected normal pressure hydrocephalus were admitted for 24 h of intraparenchymal ICP monitoring using Integra Camino (Integra LifeSciences, Saint Priest, France). The following day, an infusion test through a lumbar puncture was performed for 20 min, where 1 ml/min isotonic sterile saline was injected to gradually increase ICP. Retinal videos were recorded during these 20 min. ICP was measured once every minute. Patients with suspected VP shunt dysfunction were admitted for 24 h of intracranial pressure monitoring also using Integra Camino, and videos were captured at any given time with the intracranial pressure measurements as reference for ICP.

### Video processing and software analysis

**Figure 2:**
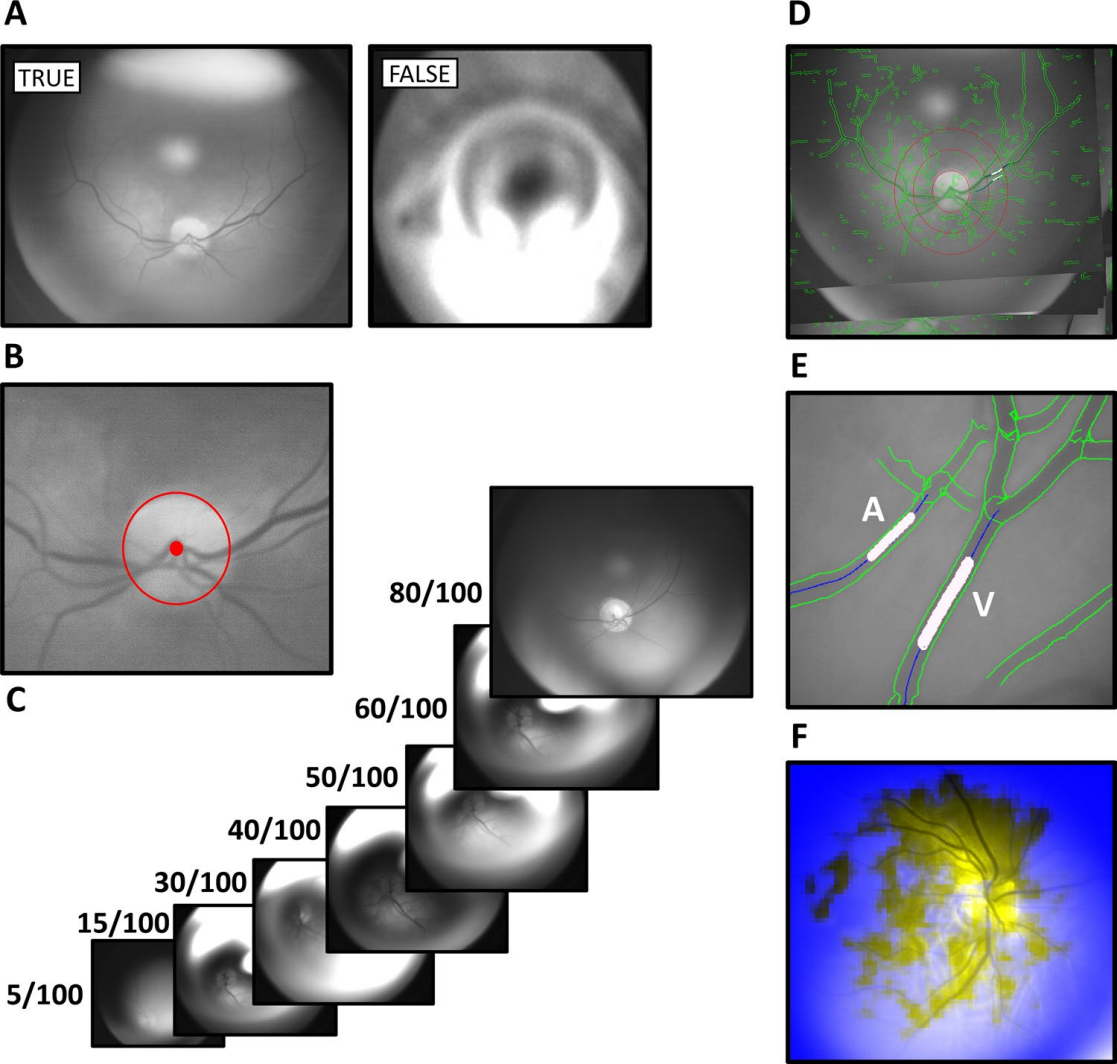
Video processing and software analysis. (**A**) ‘*Is Fundus’* function determines whether the image is that of a fundus (true) or not (false). This step excludes all non-fundus images. (**B**) The optic disc is then localized in order to measure the vessels from the same distance from the center on all images (2 radiuses). (**C**) Quality parameters filter images based on image quality (scores the image between 0–100). (**D**) Rotation: The image with the highest quality was chosen as reference, Max(Q). All images are perceived as a coordinate system (x, y, α), where α was the rotation All images were rotated to match the reference image to achieve more accurate measurements of the same points. (**E**) Twenty-five points are chosen for each vessel and the diameter in pixels is measured. (**F**) An image of how the CNTK deep learning algorithm recognizes vessels of sufficient quality on 64 × 64 px images for analysis.

The video processing and software analysis are outlined in Fig. 2*.* Videos were captured with a frame width of 1,280 pixels (px) and height of 1,024 px with 16 frames per second. Image processing software (Statumanu, Glostrup, DK) was used to split the videos into individual images. The fundus was correctly identified using the software (The “Is Fundus” function) (Fig. 2A). A Microsoft Cognitive Toolkit (CNTK) deep learning tool was applied on 192 × 192 px images (downscaled for easier management) on 52.973 individual fundus images from previous recordings not included in the study. To discard images not focusing on the fundus, the system was taught manually to distinguish fundus images from non-fundus images prior to applying the deep learning algorithm, and to correctly identify the optic disc (Fig. 2B). The developed software could either identify the disc and proceed to the next step or discard the image. Quality parameters were applied giving the images a score from 0–100 based on the optic disc, vessel clarity and overall image quality (sharpness and brightness) (Fig. 2C). The image with the highest quality was chosen as reference, Max(Q). All images were perceived as a coordinate system (x, y, α), where α was the rotation (Fig. 2D). Images were rotated to match the reference image to achieve the most accurate measurements of the same points. A paired venule and arteriole were then selected manually at a distance corresponding to two optic disc radii peripherally from the optic disk. The distinction between venule and the paired arteriole was based on the appearance with the venule being generally darker and thicker than the paired arteriole. In addition, a venule never crosses another venule, only arterioles. The software marked 25 points on either side of the marked vessel point (Fig. 2E) using Halcon software program (MVTECH, Munich, De), and a Line Gauss function^23–25^ (from the program Halcon (Stemmer Imaging A/S, DK)) was used to measure the diameter of the vessel. This process was automatized through a CNTK deep learning algorithm based on 1.043.000 images of vessels to ensure correct identification of venules and arterioles on the basis of 64 × 64 px images (Fig. 2F). Vessel diameters were transferred to Excel (Microsoft, Redmond, WA) and A/V-ratios are calculated for each given full second, which includes a total of 16 images.

### Statistical considerations

All statistical analyses were performed using Stata 16 (StataCorp LLC, College Station, Texas, USA). A mixed-effect linear regression model with random intercepts was used to assess association between mean ICP and median A/V-ratio for patients with ICP values < 15 mmHg and ≥ 15 mmHg, accounting for the repeated measurements of patients. Also, mixed-effect linear regression was performed to compare median A/V values between the groups with ICP ≤ 19 and ≥ 20 ICP. In addition, a Receiver operating characteristic (ROC) curve analysis and sensitivity/specificity analysis were performed on all A/V-ratio values ≤ 19 mmHg versus ≥ 20 mmHg to assess how well A/V-ratio predicts a potentially dangerous ICP ≥ 20 mmHg.

## Results

### Study population

The study was conducted between August 2017 and December 2017 and approved by The Regional Ethics Committee (ID S-20170038) and the The Danish Data Protection Agency (18/16,859). After signed informed consent, a total of 24 patients were included in the study. Of the 24 patients, 20 patients were suspected of normal pressure hydrocephalus (NPH) and four were suspected of ventriculoperitoneal (VP) shunt dysfunction. Two patients with suspected NPH withdrew their consent. Out of the remaining 18, six were excluded due to insufficient video imaging quality, leaving a total of twelve included patients with presumed NPH. Two of the patients with suspected VP shunt dysfunction were excluded due to insufficient video quality.

### Fundus vessel diameters and intracranial pressure

Data from all patients were plotted with ICP on the y-axis and A/V-ratio on the X-axis. ICP and corresponding A/V ratios were grouped into Low mean ICP 0–14 mmHg and Medium–High mean ICP ≥ 15 mmHg for analyses. The analyses were performed on all 14 patients with a mean of 6.1 observations per patient in total 86 total observations.

**Figure 3:**
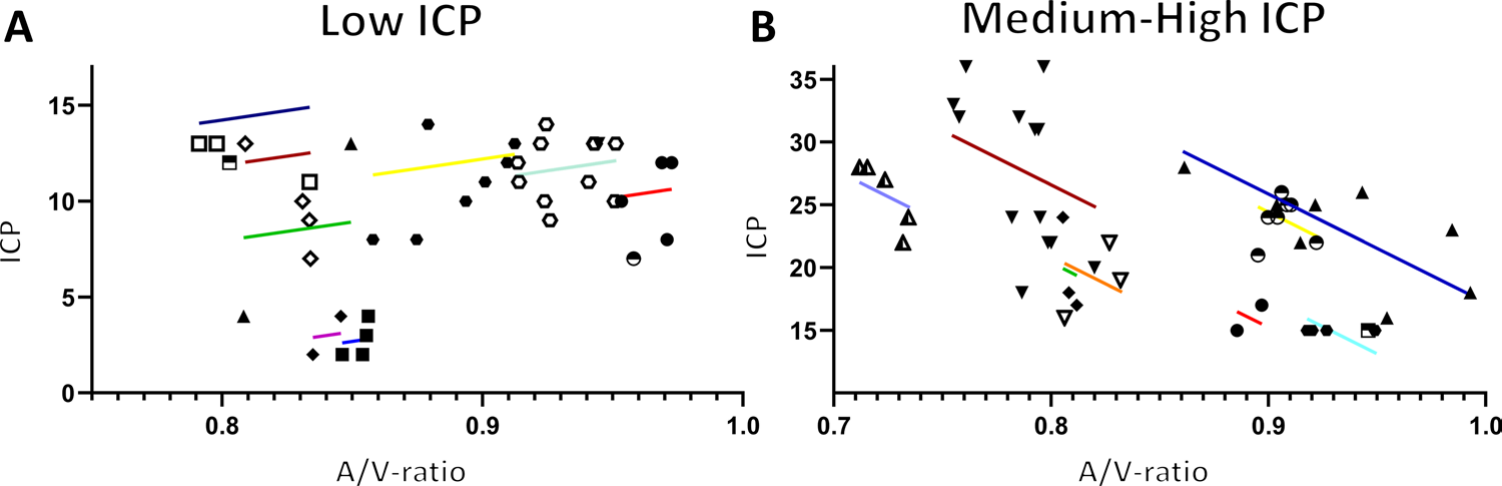
Results: Fitted data for each patient. **(A**) Graphical display of the mixed-effect linear regression of Low ICP (< 15 mmHg) based on 40 observations from 11 patients with an average of 3.6 observations per patient. Each patient is represented with a fitted line if they have two or more observations. (**B**) Same as A. except the model is based on ICP ≥ 15 mmHg (medium ICP 15–19 mmHg and high ICP ≥ 20 mmHg. Based on 46 observations from 9 patients with an average of 5.1 observations per patient. Each patient is represented with a fitted line if they have two or more observations. Some patients have observations on both sides of the ICP spectrum (Low and Medium–High).

For mean ICP values below 15 mmHg (Low ICP) the data showed an insignificant correlation between ICP and Median A/V ratio, *p* = 0.212, with a regression slope estimate of 0.0017 and 95%-CI [− 0.0010 to 0.00445] (Fig. 3A). For mean ICP equal to or above 15 mmHg the data showed a significant correlation between ICP and Median A/V ratio, *p* < 0.0001, with a slope value − 0.0023 and 95%-CI [− 0,0,035-(− 0,0011)]. The correlation between A/V-ratio and mean ICP above 15 was negative, and a positive change in ICP resulted in a fall in A/V-ratio (Fig. 3B). Further, the regression slopes for the two groups were significantly different (*p* = 0.007). Moreover, we performed a linear regression based on the A/V-ratio inter-variability between patients. The patients show a significant different A/V-ratio.

In the Medium–High ICP group, the means of the median A/V-ratios were significantly different for ICP values ≥ 20 mmHg and ICP values ≤ 19 mmHg (*p* = 0.01), with mean [95%-CI] = 0.8551 [0.8184–0.8918] for ICP values ≥ 20 mmHg and 0.8757 [0.8406–0.9107] for ICP values ≤ 19 mmHg (*p* = 0.01).

ROC curve analysis on A/V-ratio corresponding with ≥ 20 mmHg and ≤ 19 mmHg showed an area under the curve (AUC) value AUC = 0.74 (0.63–0.85), *p* = 0.0002. This analysis was used to show how well A/V-ratio predicts ICP. The sensitivity/specificity analysis with a cut-off of < 0.8015 A/V-ratio, showed a sensitivity of 94% [85–98%] and a specificity of 50% [34–66%] with a positive likelihood ratio of 9.0. This means that in a clinical setting there is a 94% chance of correctly identifying individuals with ICP ≥ 20 mmHg. However, there is also a 50% chance of identifying a healthy individual as sick. Therefore, in case a patient has a positive test, it would warrant further diagnostics such as head CT-scan or invasive ICP monitoring.

## Discussion

The present study correlated measurements of A/V-ratio in patients subjected to direct intracranial pressure monitoring. The study shows that A/V-ratio varies within the patient population. We found a correlation between a rise in ICP and a drop in A/V-ratio at mean ICP values above 15 mmHg. However, there was no correlation between ICP and A/V-ratio in low/normal ICP values (below 15 mmHg). Furthermore, potentially dangerous ICP ≥ 20 mmHg is distinguishable from not elevated ICP ≤ 19 mmHg.

Our hypothesis regarding a change in A/V-ratio and a change in ICP is supported by the recently published work by Moss et al.^26^. Moss et al. used a similar image analysis but acquired the images with scanning laser ophthalmoscopy. In the present study we used a white light fundoscopy. This results in less sharp images, but it is easier to handle for other medical personal than ophthalmologists. Despite differences in study designs the two studies come to similar conclusions. Moss et al. found no association between A/V-ratio and normal ICP. Similarly, we found no correlation at ICP lower than 15 mmHg. However, our data suggest that a significant correlation exits with ICP ≥ 15 mmHg, but with a high inter-patient variation. As shown in Fig. 3. A and B, it is clear that the patients have various A/V-ratio for the same ICP. Moss et al. conclude that there is a decrease in both arteriole and venule diameter in subjects with high ICP, and also that a short-term decrease in ICP causes the venules diameter to decrease, thereby increasing A/V-ratio. The latter corresponds well with our findings. During an infusion test to diagnose normal pressure hydrocephalus sterile saline is injected in order to raise the intracranial pressure. As ICP raises above 20 mmHg the venule distends resulting in a decrease in A/V-ratio. This response has been reported previously^27,28^.

Based on our data and the literature, we suggest that intraocular pressure (IOP) influences A/V-ratio. IOP ranges from 5–20 mmHg^29^. The vessels of the retina run within the optic nerve dural sheath^30^ and therefore are surrounded by CSF and connected to the subarachnoid space. Thus, changes in the intracranial pressure might potentially reflect the optic nerve vessel diameter^31,32^ and is affected by both changes in ICP as well as IOP^33,34^. The intraocular and intracranial spaces are both pressure compartments with variable pressures. The passing vessels are therefore regulated according to the Starling resistor mechanism,a fluid dynamic that governs the flow in collapsible tubes exposed to variable external pressure^35^. Therefore, increased pressure in one compartment will cause vessels to partially collapse, which in turn increases resistance to flow. It has long been known that IOP responds to a rise in ICP^36^, which changes the transmural equilibrium in the retinal vessels. The cerebral pressure–volume curve shows that autoregulation ensures transmural equilibrium until an ICP > 20 mmHg causes a change in the equilibrium^37^. Due to physiological differences between veins and arteries, veins are more compliant than arteries, which causes veins to distend when a rise in ICP occurs^38^.

The above corresponds with our data (ICP above 15 mmHg, medium–high), where a rise in ICP causes a drop in A/V-ratio. However, it does not explain the data from 0–15 mmHg (low). We hypothesize the data resembles some form of inverted U with the IOP as top point of A/V-ratio (highest value). However, this needs to be explored in future studies, where IOP will be included. We hypothesize that the retinal vein collapses when ICP < IOP due to forces mentioned above and by perceived distention in the 2D images. This is further supported by the works of Firsching et al.^39^, where they measured both IOP and venous outflow pressure (vOP) of the central retinal vein. They describe that the pressure within the central retinal vein is greater or equal to ICP because the vein follows the optic nerve prior to draining into the venous sinus, which is independent from IOP because the vein collapses when IOP > vOP.

Limitations: In the present study we did not measure IOP. Further studies are needed to confirm the relationship between IOP and ICP. Second, we recorded mean ICP, and we can therefore not account for changes in vein pulsation or beta-waves.

## Conclusion

In this pilot investigation, a novel methodology using a standard CE marked fundus camera for a quick, easy and inexpensive way to visualize the arterioles and venules of the eye is described. We used a semi-automatized software to measure the diameter of the vessels, and the arteriole/venule ratio was calculated. Despite limitations, a significant change in A/V-ratio could be correlated to ICP. A/V-ratio decreased with ICP above 15 mmHg (*p* = 0.0001) and there was a significant difference between ICP that is considered to be elevated and potentially dangerous (≥ 20 mmHg) and not elevated ICP. The technique shows promise for non-invasive detection of changes in ICP and possibly detection of absolute ICP values. However, further studies are needed to validate this.
